# Influence of milk microbiota on *Listeria monocytogenes* survival during cheese ripening

**DOI:** 10.1002/fsn3.1806

**Published:** 2020-07-31

**Authors:** Jeeyeon Lee, Yeongeun Seo, Jimyeong Ha, Sejeong Kim, Yukyung Choi, Hyemin Oh, Yewon Lee, Yujin Kim, Joohyun Kang, Eunyoung Park, Yohan Yoon

**Affiliations:** ^1^ Department of Food and Nutrition Dong Eui University Busan Korea; ^2^ Department of Food and Nutrition Sookmyung Women’s University Seoul Korea; ^3^ Risk Analysis Research Center Sookmyung Women’s University Seoul Korea

**Keywords:** *Listeria monocytogenes*, microbiota, pasteurized milk cheese, raw milk cheese, survival

## Abstract

This study aimed to compare the three strains of *Listeria monocytogenes* survival in raw milk cheese and pasteurized milk cheese and to suggest the effect of milk microbiota on survival. *L. monocytogenes* cell counts decreased in all cheese as ripening time increased, and the survival rate was different for the strains of *L. monocytogenes*. Furthermore, *L. monocytogenes* survived longer in raw milk cheese than in pasteurized milk cheese. The difference of bacterial survival in each cheese was independent of A_w_ or the *Lactobacillus* spp. populations in cheeses; there was no difference in A_w_ or *Lactobacillus* spp. populations in all cheeses. The richness of microbiota in raw milk was little higher than in pasteurized milk, and five phyla (Chloroflexi, Cyanobacteria, Deinococcus–Thermus, Lentisphaerae, and Verrucomicrobia) were present only in raw milk. Also, organic acid‐producing bacteria were presented more in pasteurized milk compared with raw milk; thus, the growth of *L. monocytogenes* was slower in pasteurized milk. In conclusion, differences in the microbial community of milk can affect the growth of *L. monocytogenes*. Making cheese using raw milk is a risk of *L. monocytogenes* infection; thus, efforts to prevent growth of *L. monocytogenes* such as the use of appropriate food additives are required.

## Graphical abstract

Cell counts of *L. monocytogenes* in all cheese samples were decreased as ripening time increased. Five phyla (Chloroflexi, Cyanobacteria, Deinococcus–Thermus, Lentisphaerae, and Verrucomicrobia) were only existed in raw milk; therefore, the different composition of microbiome between raw milk and pasteurized milk could be affected to the *L. monocytogenes* survival.

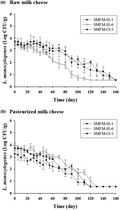

## 1 INTRODUCTION

Levels of surplus raw milk are continuously growing; in addition, the demand for cheese is currently increasing (Lee, 2017). As a result, the farmstead dairy industry is under increasing pressure to generate profits from surplus milk; however, the safety of farmstead dairy products has not been clearly verified in Korea (Lee & Yoon, 2017). Several food‐borne pathogens, such as *Listeria monocytogenes*, *Escherichia coli*, *Staphylococcus aureus*, and *Salmonella*, have been detected in cheeses (Coroneo et al., 2016; Doménech, Jimenez‐Belenguer, Amoros, Ferrus, & Escriche, 2015; Gould, Mungai, & Barton Behravesh, 2014; Iannetti et al., 2016; Jang et al., 2018; Kabuki, Kuaye, Wiedmann, & Boor, 2004; Kim, Yoo, Ham, & Oh, 2018; Kramarenko et al., 2013; Ombarak, Hinenoya, Elbagory, & Yamasaki, 2018; Papadopoulos et al., 2019; Traversa et al., 2015; Van Kessel, Karns, Gorski, McCluskey, & Perdue, 2004). Coliforms have been isolated from bulk tank milk in the United States, and, in one study, 95% of samples were found to be contaminated with coliforms (Van Kessel et al., 2004). In addition, Ombarak et al. (2018) reported that over 40% of raw milk cheese samples are contaminated with *E. coli*. Therefore, there is a genuine risk of food‐borne illness associated with cheese (Baylis, 2009; De Buyser, Dufour, Maire, & Lafarge, 2001). The prevalence of *L. monocytogenes* in cheese reported and the detection rate ranges from 2.2% to 17% (Coroneo et al., 2016; Doménech et al., 2015; Iannetti et al., 2016; Kabuki et al., 2004). Listeriosis is a food‐borne illness that can occur following consumption of cheese (Gaulin, Ramsay, & Bekal, 2012; McIntyre, Wilcott, & Naus, 2015). In northwest Switzerland, 10 cases of listeriosis were reported to be caused by the consumption of a soft cheese known as “tomme” and led to three deaths and septic abortion in two pregnant women (Bille et al., 2006).

*Listeria monocytogenes* is a zoonotic, gram‐positive, facultative anaerobe (Nho, Abdelhamed, Reddy, Karsi, & Lawrence, 2015). This pathogen is the causative agent of human listeriosis (Allerberger & Wagner, 2010), for which the mortality rate is reported to be 20%‐30% (World Health Organization, 2018). *L. monocytogenes* can survive in acidic and high salt environments and can tolerate low temperatures (Allerberger & Wagner, 2010; Lee et al., 2016). The ability of *L. monocytogenes* to survive in certain environments differs from strain to strain (Kale et al., 2017; Takahashi, Kuramoto, Miya, & Kimura, 2011). Kale et al. (2017) reported that among 104 *L. monocytogenes* strains, 13 strains could grow in a high salt environment (12.5% sodium chloride) and 22 strains showed tolerance to a low temperature (4°C). Cheese has a high salt content and a low pH and is ripened at a low temperature; nevertheless, it is possible that *L. monocytogenes* could survive in cheese, and its survival ability may vary among strains. Cheese can be made from raw milk or pasteurized milk, and the microbiota composition of raw milk and pasteurized milk may be different, which could affect the survival of *L. monocytogenes*.

Therefore, the objective of this study was to investigate the ability of different *L. monocytogenes* strains to survive in cheese made from raw or pasteurized milk and to reveal the effects of milk microbiota on the survival of *L. monocytogenes*.

## 2 MATERIALS AND METHODS

### 2.1 Inoculum preparation

Each colony of three *L. monocytogenes* strains (*L. monocytogenes* SMFM‐SI‐1, SMFM‐SI‐6, or SMFM‐CI‐1) on the plate, which were isolated from either animal carcasses or human patients (Oh et al., 2018), was inoculated into 10 ml tryptic soy broth supplemented with 0.6% yeast extract (TSBYE; Becton, Dickinson, and Company, Franklin Lakes, NJ, USA) and incubated at 30°C for 24 hr. The 0.1 ml portions of each culture were subcultured in 10 ml TSBYE and incubated at 30°C for 24 hr. Subcultures were centrifuged at 1,912 × *g* at 4°C for 15 min. Pellets were washed twice with phosphate‐buffered saline (PBS; pH 7.4; 0.2 g KH_2_PO_4_, 1.5 g Na_2_HPO_4_, 8.0 g NaCl, and 0.2 g KCl in 1 L dH_2_O) and resuspended in PBS. Each suspension of *L. monocytogenes* was serially diluted with PBS to obtain 5–6 Log CFU/ml.

### 2.2 Cheddar cheese preparation and inoculation

Three strains of *L. monocytogenes* were contaminated in raw milk and pasteurized milk, respectively, with a level of 3–4 Log CFU/ml. A total of six types of cheeses were prepared using two types of milk (raw milk and pasteurized milk) contaminated with three strains of *L. monocytogenes*. A 0.01% (w/v) mesophilic starter culture (*Lactococcus lactis* subsp. *lactis* and *L. lactis* subsp. *cremoris,* with lactose; New England Cheese Making Supply Company, South Deerfield, MA, USA) was inoculated to all *L. monocytogenes*‐contaminated milks followed by incubation at 32°C for 60 min. Liquid animal rennet (0.02% (v/v); Mysecoren300FK, MAYSA, Istanbul, Turkey) was added to the milks, which were, then, left for 30–45 min to form curd. The curd was cut into cubes (2 × 2 cm) and stirred slowly. To remove the whey, the curd was slowly heated to 38°C via a 1°C increase every 5 min. Cheddaring (the repeated process of cutting and stacking curd) was performed, and the whey was removed using a cotton cloth. NaCl (2% w/w) was added to the curd, which was mixed well before being placed into a cheese mold lined with cloth to form a block of cheese. Curd was pressed at 25°C for 40–50 min and then re‐pressed with 10 times the weight of the curd for 12 hr. The cheese was stored at 13–15°C for ripening. Cheese preparation was replicated twice.

### 2.3 Microbial analysis

Microbial analysis was performed at intervals of 5 days until 60 days of ripening and at intervals of 10 days from 70 to 160 days of ripening. The 25 g of cheese was cut aseptically from the cheese block and placed into a filter bag (3M^™^, St. Paul, MN, USA). Fifty milliliters of 0.1% buffered peptone water (BPW; Becton, Dickinson and Company) was added to the filter bag, and the bag's contents were homogenized for 60 s using a pummeller (BagMixer^®^ 400W, Interscience, St. Nom, France). Homogenates were serially diluted with 0.1% BPW, and the 0.1 ml dilutions were plated on PALCAM agar (Oxoid Ltd., Basingstoke, Hampshire, UK) with 0.4% of PALCAM supplement. The plates were incubated at 30°C for 48 hr for *L. monocytogenes* enumeration. To enumerate *Lactobacillus* spp., the dilutions were also plated on Lactobacilli MRS agar (Becton, Dickinson and Company) and incubated at 37°C for 24 hr.

### 2.4 A_w_ measurement in cheese

On the same days as the microbial analysis, the water activity (A_w_) of the cheese was measured. Cheese samples were cut into small pieces and filled with a plastic shell up to 70%. The A_w_ was then measured using an AQS‐31‐TC water activity meter (NAGY, Siedlerstrabe, Gaufelden, Germany).

### 2.5 Comparison of microbiota between raw milk and pasteurized milk

To compare the effect of microbiota on the survival of *L. monocytogenes*, next‐generation sequencing (NGS) analysis was performed to determine the microbiomes of the raw milk and pasteurized milk. DNA was extracted from the milk using a PowerSoil^®^ DNA Isolation Kit (MO BIO Laboratories, Inc., Carlsbad, CA, USA), and a sequencing library was prepared by PCR using a Nextera XT Index Kit (Illumina, San Diego, CA, USA). Sequencing was performed using the Illumina MiSeq^®^ System (Illumina) to obtain raw data. A FASTQ file was created in the MiSeq Control Software (v2.2) and bcl2fastq (v1.8.4) using the raw data, and the PhiX sequence was removed by Burrows‐Wheeler Aligner (Li & Durbin, 2009). Paired‐end data were sorted using FLASH (v1.2.11) (Magoč & Salzberg, 2011). The data were then processed to remove sequencing errors, and CD‐HIT‐OTU was used to perform clustering with more than 97% sequence similarity to obtain operational taxonomic units (OTU) (Li, Fu, Niu, Wu, & Wooley, 2012). The taxonomic assignment was performed using the BLASTN program (v2.4.0) and the NCBI 16S Microbial reference database (Zhang, Schwartz, Wagner, & Miller, 2000). Microbiota diversity was subsequently analyzed using QIIME (v1.8) (Caporaso et al., 2010).

### 2.6 Statistical analysis

The experiment to obtain the microbial survival data was replicated twice with two samples in each replicate (*n* = 4). The data were analyzed using a mixed model procedure in SAS^®^ (version 9.4; SAS Institute Inc., USA). A pairwise *t* test at α = 0.05 was used for mean comparisons.

## 3 RESULTS AND DISCUSSION

**FIGURE 1:**
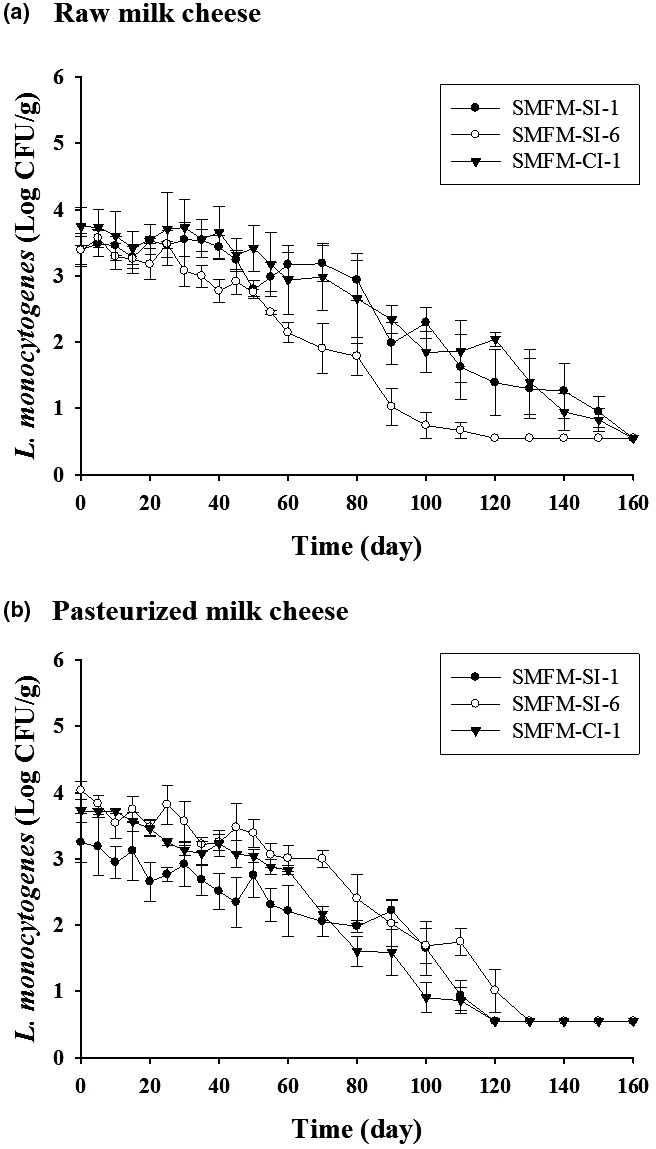
Cell counts of *Listeria monocytogenes* strains SMFM‐SI‐1, SMFM‐SI‐6, and SMFM‐CI‐1 in raw milk cheese (a) and pasteurized milk cheese (b)

*Listeria monocytogenes* cell counts decreased gradually as ripening time increased in all raw milk cheese and pasteurized milk cheese. Likewise, the initial A_w_ (0.957–0.987) of the cheeses decreased to 0.708–0.801 during ripening. When the cell counts were significantly reduced for the first time compared to the initial cell counts (*p* < .05), it was judged as “the first significant reduction.” The first significant reductions in *L. monocytogenes* cells in raw milk cheese were observed on days 90, 50, and 80 of ripening for strains SMFM‐SI‐1, SMFM‐SI‐6, and SMFM‐CI‐1, respectively (*p* < .05; Figure 1). The first significant reductions in pasteurized milk cheese were observed on days 55, 35, and 25 of ripening for strains SMFM‐SI‐1, SMFM‐SI‐6, and SMFM‐CI‐1, respectively (*p* < .05; Figure 1). These results indicate that there were variations in the abilities of different *L. monocytogenes* strains to survive in cheese. Zoz et al. (2017) reported variations in *L. monocytogenes* strain survival in a desiccated environment (75% relative humidity); of the 30 strains tested, strain Lm109 was the most viable in this environment. Similarly, Valero, Hernández, Esteban‐Carbonero, and Rodríguez‐Lázaro (2018) observed strain variations among the survival of *L. monocytogenes* in processed grated cheese, and *L. monocytogenes* LBMM1109 showed the highest level of survival. Also, *L. monocytogenes* survives better in a dehydrated environment when compared to other bacteria, such as *S. aureus* and *Salmonella* Typhimurium (Takahashi et al., 2011).

*L. monocytogenes* SMFM‐SI‐6 cell counts fell below the detection limit after 120 days of ripening, but this took 160 days for SMFM‐SI‐1 and SMFM‐CI‐1 in raw milk cheese (Figure 1). Otherwise, cell counts of three *L. monocytogenes* strains fell below the detection limit between days 120 and 130 in pasteurized milk cheese (Figure 1). As a result, the death rate of *L. monocytogenes* was slower in raw milk cheese than in pasteurized milk cheese during the ripening. We considered that these survival differences were related to differences in A_w_; however, similar A_w_ values were observed in both cheeses on day 160 of ripening (0.708–0.801). Thus, we thought that different levels of *Lactobacillus* spp. between raw milk cheese and pasteurized milk cheese influenced *L. monocytogenes* survival ability; however, the concentration of *Lactobacillus* spp. in all cheeses was maintained at 6–9 Log CFU/g, with no difference between the two types of cheese. Next, we compared the microbiomes of raw milk and pasteurized milk using NGS. The values of Inverse Simpson were 0.98 in both milk samples; therefore, there was no difference in microbiota diversity between raw milk and pasteurized milk. However, raw milk exhibited higher richness (Chao1 value = 444) than pasteurized milk (Chao 1 value = 354), indicating that more microorganisms (specifically, the five phyla Chloroflexi, Cyanobacteria, Deinococcus–Thermus, Lentisphaerae, and Verrucomicrobia) were present in raw milk (Table 1). In family level, 105 families in raw milk and 91 families in pasteurized milk were analyzed by NGS. Also, in genus level, 196 genus in raw milk and 162 genus in pasteurized milk were identified by NGS (Table 2). Based on these results, we speculated that the different compositions of these microbial communities may influence the longer survival of *L. monocytogenes* strains in raw milk cheese than in pasteurized milk cheese. Several organic acids (lactic acid, propionic acid, and acetic acid, etc.) had antimicrobial activity. In the genus level, the ratio of organic acid‐producing bacteria (*Lactobaciilus* spp. [Firmicutes phylum], *Lactococcus* spp. [Firmicutes phylum], *Streptococcus* spp. [Firmicutes phylum], *Bifidobacterium* spp. [Actinobacteria phylum], and *Roseomonas* spp. [Proteobacteria phylum]) was higher in pasteurized milk (6.49%) compared with raw milk (4.81%). Acid production may have affected the survival of *L. monocytogenes*. Wemmenhove, van Valenberg, van Hooijdonk, Wells‐Bennik, and Zwietering (2018) said that lactic acid was an inhibitor for growth of *L. monocytogenes* in Gouda cheese. There was no *Listeria* spp. in both milk. Schvartzman et al. (2011) observed that *L. monocytogenes* grows in raw milk cheese, but its survival ability is weakened in pasteurized milk cheese. They also reported that the different composition of background microflora in raw milk and pasteurized milk could affect the fate of *L. monocytogenes* (Schvartzman et al., 2011). Although not clearly identified, microbial community differences may have affected *L. monocytogenes* survival. Additionally, it was revealed that several harmful bacteria such as *Actinobaculum schaalii*, *Eubacterium moniliform*, *Flavonifractor plautii*, *Acinetobacter lwoffii*, and *Exiguobacterium aurantiacum* existed only in raw milk (Bank, Jensen, Hansen, Søby, & Prag, 2010; Berger et al., 2018; Liang, Yin, Xu, & Chen, 2017; Pitt et al., 2007; Regalado, Martin, & Antony, 2009). *F. plautii* is gram‐positive bacteria and can cause the acute cholecystitis (Berger et al., 2018). Also, *E. moniliform* and *E. aurantiacum* were bacteria isolated from patients with bacteremia (Liang et al., 2017; Pitt et al., 2007). Thus, it is considered that the possibility of outbreak is high when people intake raw milk cheese.

**TABLE 1:** Taxonomic compositions of raw milk and pasteurized milk at the phylum level

Phylum	Raw milk (%)	Pasteurized milk (%)
Firmicutes	55.64	57.47
Fusobacteria	0.21	0.04
Lentisphaerae	0.06	‐
Nitrospirae	0.09	0.17
Planctomycetes	0.17	0.14
Proteobacteria	17.37	15.30
Spirochaetes	0.84	0.47
Tenericutes	0.54	0.84
Verrucomicrobia	0.02	‐
Actinobacteria	9.22	7.48
Acidobacteria	0.07	0.20
Bacteroidetes	13.04	15.81
Chlamydiae	0.12	0.10
Chloroflexi	0.17	‐
Cyanobacteria	0.01	‐
Deinococcus–Thermus	0.01	‐
Other	2.39	1.99

**TABLE 2:** The number of microorganisms presented by level

Milk type	Phylum	Class	Order	Family	Genus	Species
Raw milk	17	31	56	105	196	312
Pasteurized milk	12	21	41	91	162	256

In conclusion, *L. monocytogenes* survival was observed to vary among strains. Furthermore, *L. monocytogenes* can survive longer in raw milk cheese than in pasteurized milk cheese during cheese ripening, and we believe that survival of *L. monocytogenes* is influenced by the differences of microbiota composition (i.e., organic acid‐producing bacteria) between the raw milk and pasteurized milk. According to the results of this study, making cheese using raw milk as it cannot guarantee safety against listeriosis. Therefore, when making cheese using raw milk, various efforts will be required, such as adding appropriate food additives (e.g. lactic acid) that can control *L. monocytogenes* to inhibit the growth of *L. monocytogenes* and further prevent *L. monocytogenes* infection.

## References

[fsn31806-bib-0001] Allerberger, F. , & Wagner, M. (2010). Listeriosis: A resurgent foodborne infection. Clinical Microbiology and Infection, 16, 16–23. 10.1111/j.1469-0691.2009.03109.x 20002687

[fsn31806-bib-0002] Bank, S. , Jensen, A. , Hansen, T. M. , Søby, K. M. , & Prag, J. (2010). Actinobaculum schaalii, a common uropathogen in elderly patients, Denmark. Emerging Infectious Diseases, 16, 76–80. 10.3201/eid1601.090761 20031046 PMC2874361

[fsn31806-bib-0003] Baylis, C. L. (2009). Raw milk and raw milk cheeses as vehicles for infection by verocytotoxin‐producing *Escherichia coli* . International Journal of Dairy Technology, 62, 293–307. 10.1111/j.1471-0307.2009.00504.x

[fsn31806-bib-0004] Berger, F. K. , Schwab, N. , Glanemann, M. , Bohle, R. M. , Gärtner, B. , & Groesdonk, H. V. (2018). *Flavonifractor* (*Eubacterium*) *plautii* bloodstream infection following acute cholecystitis. IDCases, 14, e00461 10.1016/j.idcr.2018.e00461 30425923 PMC6232644

[fsn31806-bib-0005] Bille, J. , Blanc, D. S. , Schmid, H. , Boubaker, K. , Baumgartner, A. , Siegrist, H. , … Waespi, U. (2006). Outbreak of human listeriosis associated with tome cheese in northwest Switzerland, 2005. Euro Surveillance: Bulletin Europeen Sur Les Maladies Transmissibles= European Communicable Disease Bulletin, 11, 91–93. 16801693

[fsn31806-bib-0006] Caporaso, J. G. , Kuczynski, J. , Stombaugh, J. , Bittinger, K. , Bushman, F. D. , Costello, E. K. , … Knight, R. (2010). QIIME allows analysis of high‐throughput community sequencing data. Nature Methods, 7, 335–336. 10.1038/nmeth.f.303 20383131 PMC3156573

[fsn31806-bib-0007] Coroneo, V. , Carraro, V. , Aissani, N. , Sanna, A. , Ruggeri, A. , Succa, S. , … Sanna, C. (2016). Detection of virulence genes and growth potential in *Listeria monocytogenes* strains isolated from ricotta salata cheese. Journal of Food Science, 81, M114–M120. 10.1111/1750-3841.13173 26666835

[fsn31806-bib-0008] De Buyser, M. L. , Dufour, B. , Maire, M. , & Lafarge, V. (2001). Implication of milk and milk products in food‐borne diseases in France and in different industrialised countries. International Journal of Food Microbiology, 67, 1–17. 10.1016/S0168-1605(01)00443-3 11482557

[fsn31806-bib-0009] Doménech, E. , Jimenez‐Belenguer, A. , Amoros, J. A. , Ferrus, M. A. , & Escriche, I. (2015). Prevalence and antimicrobial resistance of *Listeria monocytogenes* and *Salmonella* strains isolated in ready‐to‐eat foods in Eastern Spain. Food Control, 47, 120–125. 10.1016/j.foodcont.2014.06.043

[fsn31806-bib-0010] Gaulin, C. , Ramsay, D. , & Bekal, S. (2012). Widespread listeriosis outbreak attributable to pasteurized cheese, which led to extensive cross‐contamination affecting cheese retailers, Quebec, Canada, 2008. Journal of Food Protection, 75(1), 71–78. 10.4315/0362-028X.JFP-11-236. 22221357

[fsn31806-bib-0011] Gould, L. H. , Mungai, E. , & Barton Behravesh, C. (2014). Outbreaks attributed to cheese: Differences between outbreaks caused by unpasteurized and pasteurized dairy products, United States, 1998–2011. Foodborne Pathogens and Disease, 11, 545–551. 10.1089/fpd.2013.1650 24750119 PMC4593610

[fsn31806-bib-0012] Iannetti, L. , Acciari, V. A. , Antoci, S. , Addante, N. , Bardasi, L. , Bilei, S. , … Migliorati, G. (2016). *Listeria monocytogenes* in ready‐to‐eat foods in Italy: Prevalence of contamination at retail and characterisation of strains from meat products and cheese. Food Control, 68, 55–61. 10.1016/j.foodcont.2016.03.036

[fsn31806-bib-0013] Jang, K. , Lee, J. , Lee, H. , Kim, S. , Ha, J. , Choi, Y. , … Lee, S. (2018). Pathogenic characteristics and antibiotic resistance of bacterial isolates from farmstead cheeses. Food Science of Animal Resources, 38, 203–208. 10.5851/kosfa.2018.38.1.203 PMC5932968 29725238

[fsn31806-bib-0014] Kabuki, D. Y. , Kuaye, A. Y. , Wiedmann, M. , & Boor, K. J. (2004). Molecular subtyping and tracking of *Listeria monocytogenes* in Latin‐style fresh‐cheese processing plants. Journal of Dairy Science, 87, 2803–2812. 10.3168/jds.S0022-0302(04)73408-6 15375038

[fsn31806-bib-0015] Kale, S. B. , Kurkure, N. V. , Doijad, S. P. , Poharkar, K. V. , Garg, S. , Rawool, D. B. , & Barbuddhe, S. B. (2017). Variations in stress tolerance abilities of diverse *Listeria monocytogenes* isolates. International Journal of Current Microbiology and Applied Sciences, 6, 2246–2258. 10.20546/ijcmas.2017.605.250

[fsn31806-bib-0016] Kim, J. H. , Yoo, J. G. , Ham, J. S. , & Oh, M. H. (2018). Direct detection of *Escherichia coli*, *Staphylococcus aureus*, and *Salmonella* spp. in animal‐derived foods using a magnetic bead‐based immunoassay. Food Science of Animal Resources, 38, 727–736. 10.5851/kosfa.2018.e11 PMC6131368 30206432

[fsn31806-bib-0017] Lee,C. B. (2017). 2016 Dairy statistics yearbook. Sejong, Republic of Korea: Korea Dairy Committee.

[fsn31806-bib-0018] Kramarenko, T. , Roasto, M. , Meremäe, K. , Kuningas, M. , Põltsama, P. , & Elias, T. (2013). *Listeria monocytogenes* prevalence and serotype diversity in various foods. Food Control, 30, 24–29. 10.1016/j.foodcont.2012.06.047

[fsn31806-bib-0019] Lee, J. , Ha, J. , Kim, S. , Lee, S. , Lee, H. , Yoon, Y. , & Choi, K. H. (2016). The correlation between NaCl adaptation and heat sensitivity of *Listeria monocytogenes*, a foodborne pathogen through fresh and processed meat. Food Science of Animal Resources, 36, 469–475. 10.5851/kosfa.2016.36.4.469 PMC5018506 27621687

[fsn31806-bib-0020] Lee, J. , & Yoon, Y. (2017). Microbiological safety concerns with dairy products from farmstead plants. Journal of Milk Science and Biotechnology, 35, 215–220. 10.22424/jmsb.2017.35.4.215

[fsn31806-bib-0021] Li, H. , & Durbin, R. (2009). Fast and accurate short read alignment with Burrows‐Wheeler transform. Bioinformatics, 25, 1754–1760. 10.1093/bioinformatics/btp324 19451168 PMC2705234

[fsn31806-bib-0022] Li, W. , Fu, L. , Niu, B. , Wu, S. , & Wooley, J. (2012). Ultrafast clustering algorithms for metagenomic sequence analysis. Briefings in Bioinformatics, 13, 656–668. 10.1093/bib/bbs035 22772836 PMC3504929

[fsn31806-bib-0023] Liang, Y. , Yin, X. , Xu, J. , & Chen, S. (2017). Eubacterium moniliforme bacteremia in a woman with fractures. Clinical Laboratory, 63(10/2017), 1741–1743. 10.7754/Clin.Lab.2017.170424. 29035441

[fsn31806-bib-0024] Magoč, T. , & Salzberg, S. L. (2011). FLASH: Fast length adjustment of short reads to improve genome assemblies. Bioinformatics, 27, 2957–2963. 10.1093/bioinformatics/btr507 21903629 PMC3198573

[fsn31806-bib-0025] McIntyre, L. , Wilcott, L. , & Naus, M. (2015). Listeriosis outbreaks in British Columbia, Canada, caused by soft ripened cheese contaminated from environmental sources. BioMed Research International, 2015, 131623 10.1155/2015/131623 25918702 PMC4396127

[fsn31806-bib-0026] Nho, S. W. , Abdelhamed, H. , Reddy, S. , Karsi, A. , & Lawrence, M. L. (2015). Identification of high‐risk *Listeria monocytogenes* serotypes in lineage I (serotype 1/2a, 1/2c, 3a and 3c) using multiplex PCR. Journal of Applied Microbiology, 119, 845–852. 10.1111/jam.12876 26095922

[fsn31806-bib-0027] Oh, H. , Kim, S. , Lee, S. , Lee, H. , Ha, J. , Lee, J. , … Yoon, Y. (2018). Prevalence, serotype diversity, genotype and antibiotic resistance of *Listeria monocytogenes* isolated from carcasses and human in Korea. Food Science of Animal Resources, 38, 851–865. 10.5851/kosfa.2018.e5 PMC6238023 30479494

[fsn31806-bib-0028] Ombarak, R. A. , Hinenoya, A. , Elbagory, A. R. M. , & Yamasaki, S. (2018). Prevalence and molecular characterization of antimicrobial resistance in *Escherichia coli* isolated from raw milk and raw milk cheese in Egypt. Journal of Food Protection, 81, 226–232. 10.4315/0362-028X.JFP-17-277 29323530

[fsn31806-bib-0029] Papadopoulos, P. , Papadopoulos, T. , Angelidis, A. S. , Kotzamanidis, C. , Zdragas, A. , Papa, A. , … Sergelidis, D. (2019). Prevalence, antimicrobial susceptibility and characterization of *Staphylococcus aureus* and methicillin‐resistant *Staphylococcus aureus* isolated from dairy industries in north‐central and north‐eastern Greece. International Journal of Food Microbiology, 291, 35–41. 10.1016/j.ijfoodmicro.2018.11.007 30445283

[fsn31806-bib-0030] Pitt, T. L. , Malnick, H. , Shah, J. , Chattaway, M. A. , Keys, C. J. , Cooke, F. J. , & Shah, H. N. (2007). Characterisation of Exiguobacterium aurantiacum isolates from blood cultures of six patients. Clinical Microbiology and Infection, 13, 946–948. 10.1111/j.1469-0691.2007.01779.x 17645563

[fsn31806-bib-0031] Regalado, N. G. , Martin, G. , & Antony, S. J. (2009). *Acinetobacter lwoffii*: Bacteremia associated with acute gastroenteritis. Travel Medicine and Infectious Disease, 7, 316–317. 10.1016/j.tmaid.2009.06.001 19747669

[fsn31806-bib-0032] Schvartzman, M. S. , Maffre, A. , Tenenhaus‐Aziza, F. , Sanaa, M. , Butler, F. , & Jordan, K. (2011). Modelling the fate of *Listeria monocytogenes* during manufacture and ripening of smeared cheese made with pasteurised or raw milk. International Journal of Food Microbiology, 145, S31–S38. 10.1016/j.ijfoodmicro.2010.11.032 21176989

[fsn31806-bib-0033] Takahashi, H. , Kuramoto, S. , Miya, S. , & Kimura, B. (2011). Desiccation survival of *Listeria monocytogenes* and other potential foodborne pathogens on stainless steel surfaces is affected by different food soils. Food Control, 22, 633–637. 10.1016/j.foodcont.2010.09.003

[fsn31806-bib-0034] Traversa, A. , Gariano, G. R. , Gallina, S. , Bianchi, D. M. , Orusa, R. , Domenis, L. , … Decastelli, L. (2015). Methicillin resistance in *Staphylococcus aureus* strains isolated from food and wild animal carcasses in Italy. Food Microbiology, 52, 154–158. 10.1016/j.fm.2015.07.012 26338130

[fsn31806-bib-0035] Valero, A. , Hernández, M. , Esteban‐Carbonero, Ó. , & Rodríguez‐Lázaro, D. (2018). Modelling the fate and serogroup variability of persistent *Listeria monocytogenes* strains on grated cheese at different storage temperatures. International Journal of Food Microbiology, 286, 48–54. 10.1016/j.ijfoodmicro.2018.07.021 30036729

[fsn31806-bib-0036] Van Kessel, J. S. , Karns, J. S. , Gorski, L. , McCluskey, B. J. , & Perdue, M. L. (2004). Prevalence of Salmonellae, *Listeria monocytogenes*, and fecal coliforms in bulk tank milk on US dairies. Journal of Dairy Science, 87, 2822–2830. 10.3168/jds.S0022-0302(04)73410-4 15375040

[fsn31806-bib-0037] Wemmenhove, E. , van Valenberg, H. J. F. , van Hooijdonk, A. C. M. , Wells‐Bennik, M. H. J. , & Zwietering, M. H. (2018). Factors that inhibit growth of *Listeria monocytogenes* in nature‐ripened Gouda cheese: A major role for undissociated lactic acid. Food Control, 84, 413–418. 10.1016/j.foodcont.2017.08.028

[fsn31806-bib-0038] World Health Organization (2018). Listeriosis. Retrieved from https://www.who.int/news‐room/fact‐sheets/detail/listeriosis

[fsn31806-bib-0039] Zhang, Z. , Schwartz, S. , Wagner, L. , & Miller, W. (2000). A greedy algorithm for aligning DNA sequences. Journal of Computational Biology, 7, 203–214. 10.1089/10665270050081478 10890397

[fsn31806-bib-0040] Zoz, F. , Grandvalet, C. , Lang, E. , Iaconelli, C. , Gervais, P. , Firmesse, O. , … Beney, L. (2017). *Listeria monocytogenes* ability to survive desiccation: Influence of serotype, origin, virulence, and genotype. International Journal of Food Microbiology, 248, 82–89. 10.1016/j.ijfoodmicro.2017.02.010 28288399

